# Skeletonized mean diffusivity and neuropsychological performance in relapsing‐remitting multiple sclerosis

**DOI:** 10.1002/brb3.2591

**Published:** 2022-05-13

**Authors:** Magdalena Chylińska, Bartosz Karaszewski, Jakub Komendziński, Adam Wyszomirski, Agnieszka Sabisz, Marek Halas, Edyta Szurowska

**Affiliations:** ^1^ Department of Adult Neurology Medical University of Gdańsk, Faculty of Medicine Gdańsk Poland; ^2^ 2nd Department of Radiology Medical University of Gdańsk, Faculty of Medicine Gdańsk Poland

**Keywords:** cognition, DTI, MRI, multiple sclerosis, PSMD

## Abstract

**Background:**

Peak width of Skeletonized Mean Diffusivity (PSMD), as a novel marker of white matter (WM) microstructure damage, is associated with cognitive decline in several WM pathologies (i.e., small vessel disorders). We hypothesized that markers combining alterations in whole WM could be associated with cognitive dysfunction in relapsing‐remitting multiple sclerosis (RRMS) patients.

**Methods:**

We used PSMD based on tract‐based spatial statistics (TBSS) of diffusion tensor imaging (DTI) magnetic resonance (MR) scans. We investigated RRMS patients (*n* = 73) undergoing interferon beta (IFN‐β) therapy. In this cross‐sectional study, we investigated the association between neuropsychological data and clinical and MRI variables: PSMD, WM hypointensities, and normalized brain volume (NBV).

**Results:**

In our cohort, 37 (50.7%) patients were recognized as cognitively impaired (CI) and 36 (49.3%) patients were cognitively normal (CN). In regression analysis, PSMD was a statistically significant contributor in the California Verbal Learning Test (CVLT) list A (*p* = 0.04) and semantic fluency (*p* = 0.036). PSMD (*p* < 0.001, *r* ^2 ^= 0.35), NBV (*p* = 0.002, *r* ^2 ^= 2.6) and WM hypointensities (*p* < 0.001, *r* ^2 ^= 0.40) were major contributors to upper extremity disability (9HPT) in the CN subgroup. A significant contributor in the majority of neuropsychological measures was education attainment.

**Conclusion:**

We investigated PSMD as a new parameter of WM microstructure damage that is a contributor in complex cognitive tasks, CVLT performance, and semantic fluency. PSMD was a statistically significant contributor to upper extremity disability (9HPT) together with WM hypointensities and NBV. Education attainment proved to be relevant in the majority of cognitive domains. Further studies are needed to estimate PSMD relevance as a marker of CI in MS.

## Graphical abstract

We investigated the association between neuropsychological data and clinical and MRI variables: Peak width of Skeletonized Mean Diffusivity (PSMD), WM hypointensities and normalized brain volume. PSMD was a significant contributor in the California Verbal Learning Test list A and semantic fluency. PSMD, normalized brain volume and white matter hypointensities were major contributors to upper extremity disability (9HPT) in the CN subgroup. A significant contributor in the majority of neuropsychological measures was education attainment.

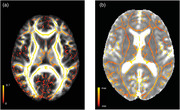

## 1 INTRODUCTION

Multiple sclerosis (MS) is a neuroinflammatory and neurodegenerative condition of the central nervous system (CNS), in which inflammation, demyelination, and axonal loss lead to the progression of disability. There are known biomarkers of white matter (WM) pathology in MS such as lesion volume (LV), lesion number, and persistent T1‐hypointense lesions (black holes) (Sahraian et al., 2009), which correlate with functional disability (Popescu et al., 2013) and CI (Rovaris et al., 2006). However, WM pathology, detectable by MRI, only partially contributes to disability and cognitive deficits in MS patients (Rocca et al., 2015). The altered microstructure of normal appearing white matter (NAWM) and normal appearing gray matter (NAGM) is beyond the scope of conventional MRI techniques but is presumed to be associated with greater physical disability and CI (Rocca et al., 2015). MR diffusion tensor imaging (DTI)‐based studies are a sensitive method for the detection of abnormalities within NAWM in MS patients (Werring et al., 1999). Numerous studies have shown increases in mean diffusivity (MD) and a reduction in fractional anisotropy (FA) associated with focal WM lesions and NAWM among patients with MS (Miller et al., 2014). There is also evidence that an alteration of the diffusion parameters may precede the formation of WM lesions (Werring et al., 2000). Furthermore, diffusion tensor MRI studies in MS exhibited additional lesion‐independent substrates of CI (Roosendaal et al., 2009). Previous DTI MR studies in the MS population revealed inconsistent results. The authors used different methods of DTI analysis in cross‐sectional and longitudinal studies.

In our study, we use a novel marker of the integrity of WM microstructure based on DTI and the skeletonization of WM tracts—PSMD (Baykara et al., 2016). PSMD showed clinical relevance as a biomarker of WM pathology and cognitive performance in a population of patients with small vessel disease (SVD) such as cerebral autosomal‐dominant arteriopathy with subcortical infarcts and leukoencephalopathy (CADASIL), cerebral amyloid angiopathy (CAA), or Alzheimer's disease (AD) (Baykara et al., 2016; McCreary, 2020). CI is widespread in the MS patient population (43–70%), affecting mainly a few predominant cognitive domains: Information processing efficiency, attention, episodic and long‐term memory, processing speed, and executive functions including verbal fluency and word list generation (Chiaravalloti & DeLuca, 2008; Rocca et al., 2015). Previous studies have already suggested that PSMD might be a reliable marker of altered cognition in MS, especially in relation to the dysfunction of information processing speed (Vinciguerra et al., 2020). This is a cross‐sectional study revealing that PSMD might be used to assess microstructural abnormalities of WM as a background of cognitive impairment in MS. In order to avoid the influence of drugs (various mechanisms of action) on the MRI diffusion parameters, unlike in other studies, herein one of the inclusion criteria was the same immunomodulatory therapy (IMT) across the whole patient sample (interferon beta [IFN‐β] therapy). Similar to previous data (Vinciguerra et al., 2020), we used a combined method of TBSS “skeletonization” of WM tracts with an analysis of diffusion histograms (Baykara et al., 2016). The combination of these techniques has two main advantages: First, the reduction of DTI data regarding contamination with cerebrospinal fluid (CSF) and other nonbrain structures (Smith et al., 2006); second, the analysis of the whole WM microstructure including NAWM. The process of skeletonization is directed at the analysis of the MD of the main fiber tracts of hemispheres that overcome the contamination of whole brain MD data through CSF (Baykara et al., 2016), comprising both affected and NAWM tracts. Histogram analysis of skeletonized MD is an appropriate method for WM diffuse pathology in MS, quantifying the total disease burden, because it captures the distribution of water molecule diffusivity obtained from the center of the main WM tracts (Deary et al., 2019). Contrary to DTI measures consisting of FA and MD metrics of WM in the selected brain region of interest, PSMD reveals the integrity of the whole WM skeleton. Moreover, PSMD as an automatic method omits many postprocessing steps and subjective operation errors (Wei et al., 2019). This quantitative, easy‐to‐implement method reflects the disease burden that could be applied to a large sample (Baykara et al., 2016). The aforementioned advantages of PSMD prompt us to assess its utility as a radiological marker of cognitive dysfunctions in major domains, in an RRMS population, and with each patient treated with the same IMT.

## 2 MATERIALS AND METHODS

### 2.1 Population

Initially, 80 patients of the Department of Adult Neurology, at the Medical University Center in Gdańsk and the University Clinical Center, Poland, who were diagnosed RRMS according to the 2010 McDonald criteria (Polman et al., 2011), were included in the study prospectively between December 2015 and December 2018. All participants had a negative history of corticosteroid use during 30 days prior to MRI data acquisition. Only patients treated with IFN‐β were included in the study. Patients with no previous switching to another disease modifying therapy were included in the study. Patients with noncompensated metabolic and systemic disorders were excluded from the study. Patients with an intake of psychoactive drugs or with diagnosed psychiatric conditions were excluded from the study. Six patients were excluded from the study due to severe depression symptoms in BDI‐II.

Seven patients were excluded from the analysis due to the insufficient quality of MRI images, mainly due to motion artifacts. Finally, 73 patients were included in the analysis.

The study protocol was approved by the local bioethics committee (Medical University of Gdańsk, Bioethics Committee). Written informed consent was obtained from all subjects participating in the study.

### 2.2 Neurological tests

Neurological assessments: The Expanded Disability Status Scale (EDSS), and the Multiple Sclerosis Functional Composite (MSFC) were performed on each patient by the same neurologist experienced in MS and included:

Timed‐25 foot walk (T25FW)—The ambulation time (in seconds) needed to walk the distance of 25 feet. The result is the average score of two 25‐foot timed walk trials (Fischer et al., 2001).

Paced auditory serial additive test (PASAT)—A measure of auditory information processing and attention: The task involves the consecutive adding of 60 pairs of digits presented in a series with 3‐s intervals (each digit is added to the preceding one); the final score is the number of correct responses (Fischer et al., 2001).

9 hole peg test (9HPT)—A quantitative measure of upper extremity (arm and hand) function. The time (in seconds) necessary to insert 9 pegs into holes and remove them using one hand is measured. The result is the average score from four trials on the 9‐HPT (two trials for each hand are averaged, converted to reciprocals of the mean time for each hand and then the two reciprocals are averaged) (Fischer et al., 2001).

### 2.3 Neuropsychological assessments

An experienced neuropsychologist performed an assessment of selected cognitive domains in all the enrolled patients, blind to important clinical and neuroimaging information. The following tests were performed: SDMT—A written version (Smith, 1982; Vogel et al., 2013), CVLT (Łojek et al., 2010; Delis et al., 2000), Wisconsin card sorting test (WCST) (Heaton et al., 1993; Jaworowska, 2002)—The Polish adaptation (Jaworowska, 2002), Benton visual retention test (BVRT)—The Polish adaptation (Jaworowska, 2013), color trails test (CTT) (D'Elia et al., 1996; Louis et al., 2012), verbal fluency test (VFT)—The Polish version (Piskunowicz et al., 2013; Vlaar & Wade, 2003), Beck depression inventory‐II (BDI‐II)—The Polish version of the questionnaire (Beck et al., 1996; Benedict et al., 2003), modified fatique impact scale (MFIS)—The Polish version of the questionnaire (Larson, 2013). A detailed description of these neuropsychological tests is provided in Supplement 1.

In the study population, two groups: Cognitively impaired (CI) and cognitively normal (CN) were distinguished. Patients were classified as CI when the results of at least two cognitive tests were abnormal. The test results of VFT (Piskunowicz et al., 2013), CVLT (Łojek et al., 2010), WCST (Jaworowska, 2013), CTT (Louis et al., 2012), and BVRT (Jaworowska, 2013) were considered abnormal when they were below the Polish limit for a given age and education, SDMT (Smith, 1982) results below 1SD were acknowledged abnormal, and PASAT‐3′ (Fischer et al., 2001) results below −1 of *Z*‐score were considered abnormal.

### 2.4 MRI data acquisition

The patients underwent MRI on the same day as a clinical examination. MRI examinations were performed with a 1.5 T scanner (Siemens Magnetom Aera) using a 20‐channel head/neck coil. The MRI protocol included the standard brain protocol for MS patients (sequences: T1‐weighted 3D, FLAIR 3D, DIR 3D, SWI, DWI, T2‐weighted sagittal and axial orientation, T1‐weighted 3D—10 min after the injection of the contrast medium) and DTI. The analysis in this project was performed with T1‐weighted 3D MPRAGE sequence and DTI; the parameters were as follows: T1‐weighted MPRAGE sequence (transverse orientation, TR = 1800 ms, TE = 3.3 ms, TI = 1000 ms, voxel size 1.4 mm × 1.4 mm × 1.1 mm, FOV 270 mm × 270 mm, slices 144, NSA = 1) and DTI (axial plane without angulation, TR = 6500 ms, TE = 85 ms, matrix 114 × 114, FOV 260 mm × 260 mm, voxel size 2.28 mm × 2.28 mm × 3.0 mm, 50 slices, slice thickness 3 mm, 3 volumes acquired with *b* = 0 s/mm^2^ and 3 repetitions of 30 diffusion gradient directions acquired with *b* = 1000 s/mm^2^.

### 2.5 MRI data analysis

T1‐weighted images and DTI images were converted to nii format by MRI Convert (https://lcni.uoregon.edu/downloads/mriconvert). Volumes of brain structures and the cortical thickness were measured by the freely available software FreeSurfer, version 6.0 (http://surfer.nmr.mgh.harvard.edu) (Fischl et al. 1999). The standard FreeSurfer processing stream recon‐all was used. Data were visually inspected. The volumes obtained from analyses were normalized to the estimated total intracranial volume (eTIV).

**FIGURE 1:**
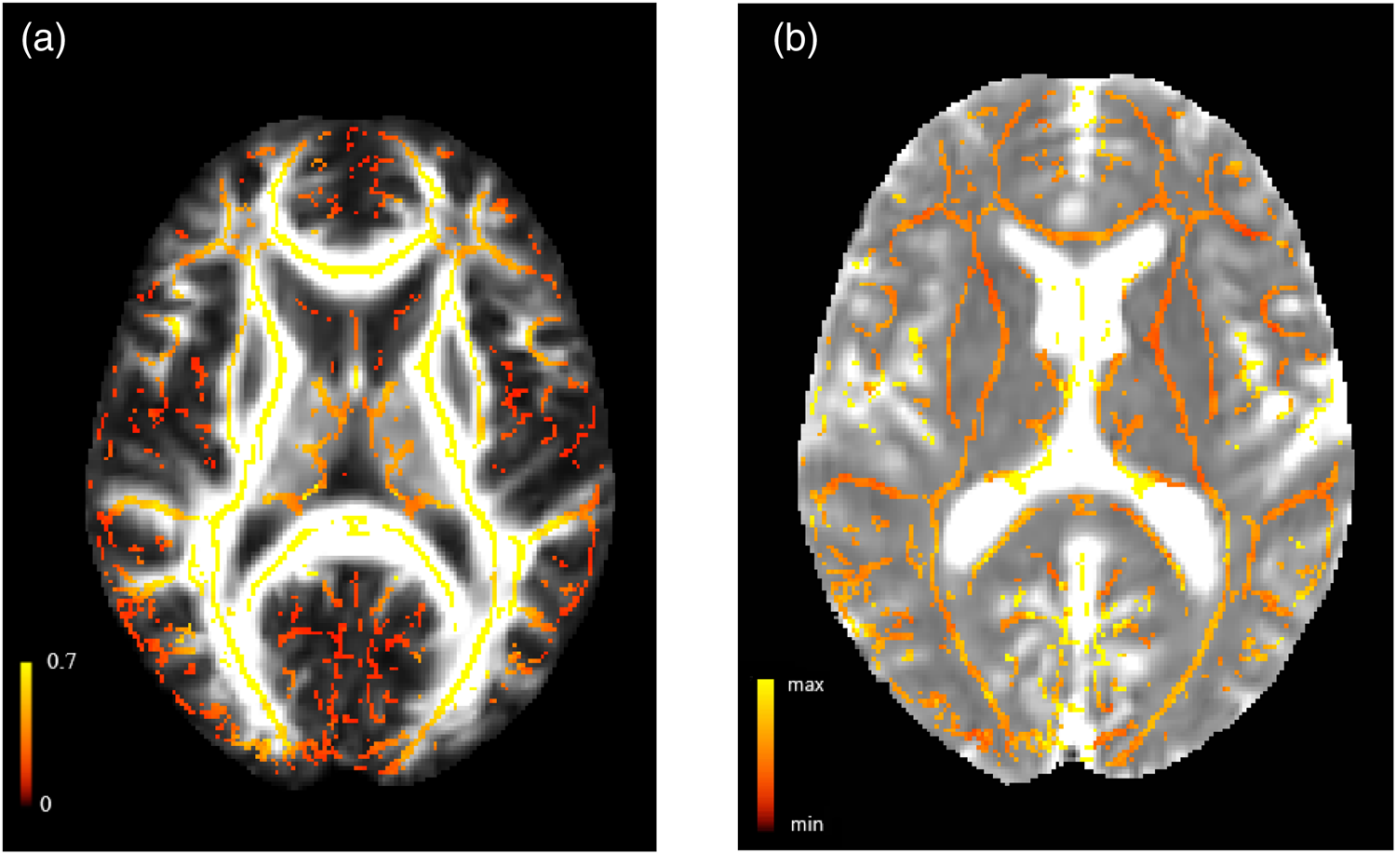
A. Example of an FA map with the superimposed skeletonized FA data of an RRMS patient. The images prepared are based on the tract‐based spatial statistics procedure in the step before the calculation of PSMD values. **B**. Example of an MD map with the superimposed skeletonized MD data of an RRMS patient. The images prepared are based on the tract‐based spatial statistics procedure in the step before the calculation of PSMD values

The quality of diffusion tensor images was checked. The number of outliers in the model and the motion were inspected in Explore DTI software. Patients whose rotation and movement were more than 1 degree and 1 mm, respectively, were excluded from the analysis. Diffusion images were preprocessed using the FreeSurfer script TRACULA (Yendiki, 2011). The processing steps included: Correction for distortions due to head motion and eddy currents, and the calculation of diffusion parameter maps (FA, MD, RD, AD). In the next step, PSMD was calculated with the PSMD tool provided at ^http://www.psmd‐marker.com9^ based on FA and MD maps. First, DTI data were skeletonized using the TBSS, part of the functional magnetic resonance imaging of the brain (FMRIB) software library (FSL) (http://www.fmrib.ox.ac.uk/fsl) and the FMRIB 1 mm FA template (at an FA threshold value of 0.2). The MD images were projected onto the skeleton using the FA derived projection parameters. In order to avoid contamination of the skeleton through CSF partial volume effects, the MD skeletons were further masked with a standard skeleton at an FA threshold value of 0.3, and a mask provided with the PSMD tool in order to exclude regions adjacent to the ventricles, such as the fornix (Figure 1). Finally, PSMD was calculated as the difference between the 95th and 5th percentiles of the MD voxel values within the WM skeleton (Baykara et al., 2016; Vinciguerra et al., 2019).

### 2.6 Statistical analysis

The mean and standard deviations or quartiles were presented for the demographic, clinical, and radiological data. Categorical variables were reported as counts and percentages. Spearman's rank correlations were determined between PSMD and clinical variables including EDSS scores, disease durations, BDI and MFIS scores, and neuropsychological test results. The rho correlation coefficient was calculated. A *p*‐value of less than 0.05 was considered to be significant. A statistical analysis was performed using the R Project for Statistical Computing R version 3.6.3 (Holding the Windsock), a language and environment for statistical computing, R Foundation for Statistical Computing, Vienna, Austria (https://www.r‐project.org/) [Chambers(2008)]. The correlation coefficient and *p*‐value were calculated using the “Hmisc” Package, August 11, 2020, Version 4.4 https://CRAN.R‐project.org/package=Hmisc.

We applied the linear regression method to assess the relationship between neuropsychological data and sets of clinical and radiological variables. We used 11 models explaining neuropsychological measures (Table 1). The clinical variables included in the models were: Age, gender, education, and disease duration. The radiological variables included in the models were: PSMD, WM hypointensities, NBV. The results from the regression models were expressed as beta coefficients with 95% confidence intervals. The coefficient of variation (*R*‐squared) was used to assess the goodness of fit statistical model. Detailed data of the multivariate linear regression analysis were included in Supplement 2. The *R*‐squared and beta coefficient were adjusted to age, disease duration, gender, and education duration. No formal adjustment for multiple testing was carried out.

**TABLE 1:** Multivariate linear regression models explaining neuropsychological data

Linear regression models	Clinical data	Demographic and clinical data	Radiological data
Model 1	SDMT	Age	PSMD
Model 2	PASAT	Gender	WM hypointensities
Model 3	VFT,phonological fluency	Education	NBV
Model 4	VFT,semantic fluency	Disease duration	
Model 5	CCT1 time		
Model 6	CCT2 time		
Model 7	CVLT list‐A		
Model 8	CVLT list‐B		
Model 9	WCST, total errors		
Model 10	WCST, percentage of conceptual responses		
Model 11	BVRT, correct total		
Model 12	EDSS		
Model 13	9 HPT		

Abbreviations: SDMT, symbol digit modalities test; PASAT, paced auditory serial additive test; VFT, verbal fluency test; CTT, color trails test; BVRT, Benton visual retention test; WCST, Wisconsin card sorting test; CVLT, California verbal learning test total recall; EDSS, the expanded disability status scale; 9 HPT, 9 hole peg test; PSMD, Peak width of skeletonized mean diffusivity; WM, white matter; NBV, normalized brain volume.

We also performed an additional univariate linear regression analysis of clinical and cognitive data and radiological measures in the two subgroups of patients: CN and CI.

## 3 RESULTS

### 3.1 Demographic and clinical characteristics

Totally, 73 patients with RRMS were studied. The group of patients with RRMS was heterogeneous, both in terms of disability (EDSS) and the duration of the disease.

The demographic and clinical characteristics of the patients are provided in Table 2. Six subjects were diagnosed with treated thyroid disease. In BDI‐II, we found 60 patients with minimal (0–13), 11 patients with mild (14–19), and 2 patients with moderate (20–28) depressive symptoms. In our cohort, 37 (50.7%) patients were recognized as CI and 36 (49.3%) patients were CN (Supplement 3, Table 1). MS subgroups did not differ statistically in terms of PSMD, NBV, and WM hypointensities, age, EDSS, and 9HPT score (Supplement 3, Table 1). We observed a statistically significant difference in education duration and cognitive test results (Supplement 3, Tables 1 and 2).

**TABLE 2:** Demographic, clinical and radiological characteristics

Characteristics, mean (SD)	Values
Number of patients	73
Gender F/M, F (%)	53/20 (62%)
Age (years)	38.5 (9.2)
Disease duration (years since diagnosis), median (Q1,Q3)	8.1 (4.0,11.0)
Education (years)	15.1 (2.4)
EDSS	2.4 (1.0)
SDMT	46,99 (11.4)
Z‐cognitive (PASAT)	0.023 (0.007)
Z‐leg average (T25FW)	0.05 (0.014)
Z‐arm average (9HPT)	0.038 (0.007)
VFT phonological fluency total	15.86 (4.98)
VFT semantic fluency total	20.64 (5.75)
CVLT Total list A	7.13 (1.97)
CVLT Total list B	5.69 (1.6)
CTT1 competition time	40.64 (16.7)
CTT2 competition time	84.17 (26.2)
WCST total errors median (Q1;Q3)	27.6 (10; 46)
WCST percentage of conceptual responses median (Q1;Q3)	15.51 (6; 21)
BVRT correct total	7.55 (1.4)
BVRT errors	3.65 (2.43)
BDI‐II	12.9 (8.6)
MFIS	28 (14.3)
PSMD	2.814 × 10^−4^ (6.96 × 10^−5^)
NBV	7.522 × 10^−1^ (3.5 × 10^−2^)
WM hypointensities vol. [normalized to eTIV]	3.092 × 10^−3^ (2.59 × 10^−3^)

Abbreviations: F, female; M, male; SD, standard deviation; EDSS, expanded disability status scale; SDMT, symbol digit modalities test; PASAT, paced auditory serial additive test; T25FW, Timed‐25 foot walk; 9HPT, 9 hole peg test; VFT, verbal fluency test; CTT, color trails test; BVRT, Benton visual retention test; WCST, Wisconsin card sorting test; CVLT TOT, California verbal learning test total recall; BDI II, Beck depression inventory; MS, multiple sclerosis; RRMS, relapsing‐remitting multiple sclerosis; SD, standard deviation; Q1, the first quartile; Q3, the third quartile; PSMD, peak width of skeletonized mean diffusivity; NBV, normalized brain volume; vol, volume; eTIV, estimated total intracranial volume.

### 3.2 Correlation between PSMD and clinical variables

**FIGURE 2:**
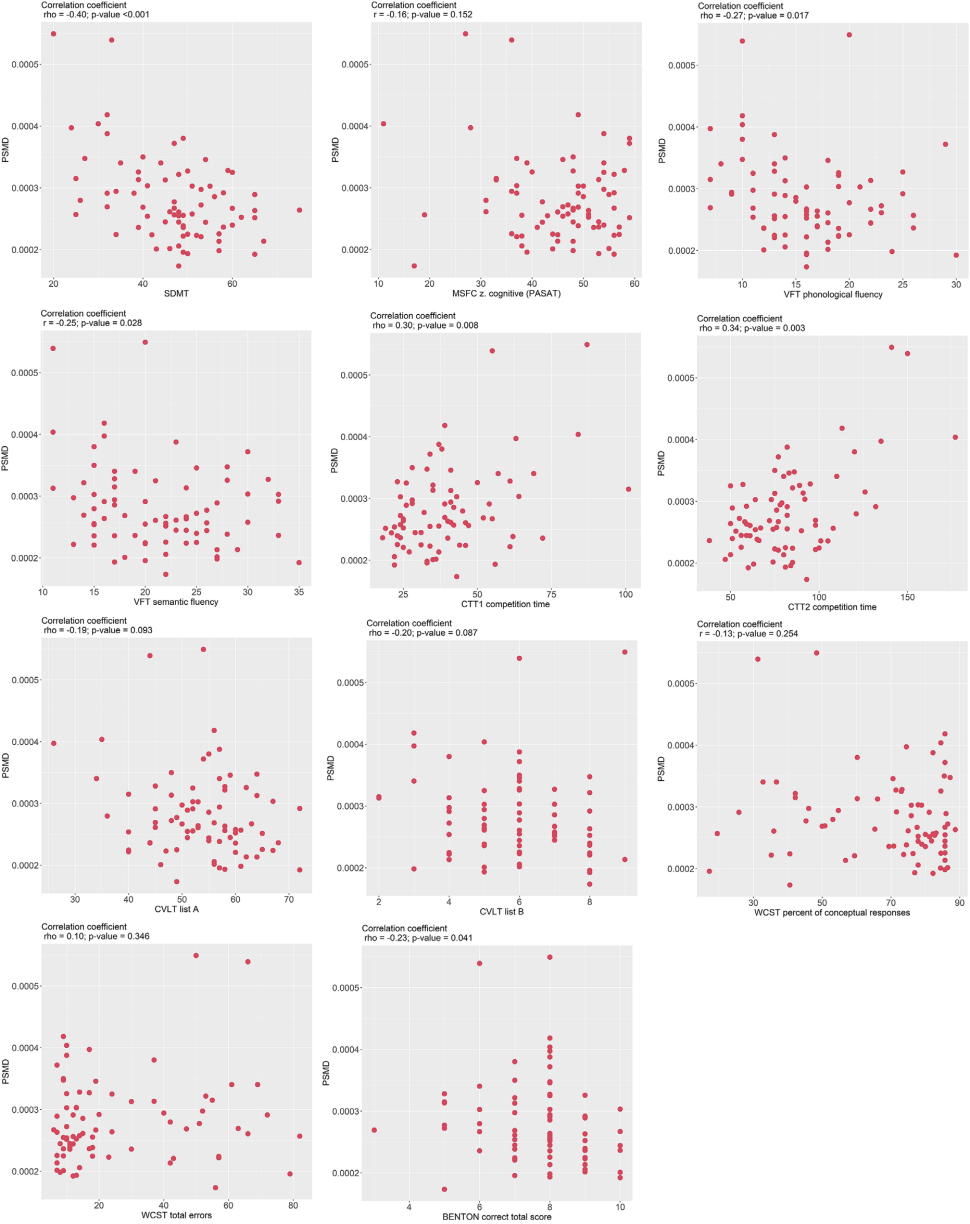
Correlation between PSMD and neuropsychological measures

In the studied RRMS population, the mean values (Table 2) of PSMD, NBV, and WM hypointensities were obtained. For clarity in understanding the results reported in Table 2, the more “affected brain tissue” had larger values of PSMD and WM hypointensities and lower NBV values. Results of the Spearman test correlation between clinical variables, neuropsychological measures, and PSMD are included in Table 3. In the studied cohort, the disease duration and the total number of relapses strongly correlated with PSMD. A moderate association was found between PSMD and the severity of the neurological disability measured with EDSS, out of which visual, mental, and cerebellar system functions were significant (*p* < 0.05). There was a moderate correlation between PSMD and upper extremity function assessed in the 9‐HP test (*p* = 0.003). Figure 2 demonstrates correlations between PSMD and neuropsychological measures, tests measuring attention, processing speed, visual scanning, language learning, and working memory. In particular, PSMD significantly correlated with CCT variables: Total time in CCT1 (*p* < 0.05) and CCT2 (*p* < 0.001) and the number of errors (*p* < 0.05). SDMT had a much stronger correlation with PSMD (*p* < 0.0001) than PASAT 3. The verbal fluency test exhibited a moderate correlation with PSMD, in two domains: Phonological fluency (*p* = 0.017) and semantic fluency (*p* = 0.048). The Benton visual retention test, assessing visual perception and visual memory, showed a moderate and significant correlation with PSMD (*p *< 0.05). We found a very weak, not significant correlation between PSMD and the results of WSCT and CVLT.

**TABLE 3:** Correlation between clinical variables and PSMD

Clinical variables	PSMD rho	*p*‐value
Disease duration	**0.31**	**0.008**
Total n of relapses	**0.37**	**0.001**
MSFC Score	−0.14	0.771
Z. leg average (T25FW)	−0.08	0.459
Z. arm average (9HPT)	**−0.33**	**0.003**
EDSS	**0.24**	**0.049**
Visual FS	**0.31**	**0.006**
Brainstem FS	0.15	0.295
Pyramidal FS	0.12	0.326
Cerebellar FS	**0.32**	**0.005**
Sensory FS	0.08	0.635
Bowel FS	0.15	0.217
Cerebral FS	**0.23**	**0.048**

Abbreviations: PSMD, peak width of skeletonized mean diffusivity; Rho, correlation coefficient Spearman's test; p≤0.05 statistically significant in bold; MSFC, multiple sclerosis functional composite; T25FW, Timed‐25 foot walk; 9HPT, 9 hole peg test, EDSS, expanded disability status scale; FS, functional system; ns, not significant.

### 3.3 Multivariate linear regression analysis

We performed a multivariate linear regression analysis, testing 11 models to explain the main neuropsychological data.

In model 1: The SDMT score was over 36% explained by the clinical and radiological variables used in the model but only education duration was a statistically significant variable (*p* < 0.001).

In models 2 and 3: The clinical and radiological variables explained only about 2% of the archived PASAT score and VFT phonological fluency score. None of the used variables were statistically significant.

In model 4: The clinical and MRI variables explained about 13% of the archived score in VFT and semantic fluency, and education duration was a statistically significant variable (*p* < 0.001).

In model 5: The clinical and MRI variables explained over 23% of the archived CTT1 score, and education duration was a statistically significant variable (*p* = 0.028).

In model 6: The clinical and MRI variables explained 20% of the archived CTT2 score and education duration was a statistically significant variable (*p* = 0.012).

In model 7: The clinical and MRI variables explained 32% of the archived CVLT list A score, and of statistical significance were education duration (*p* < 0.001), disease duration (*p* = 0.005), WM hypointensities (*p* = 0.021), and PSMD (*p* = 0.047).

In model 8: The clinical and MRI variables explained over 10% of the archived CVLT list B score, and education duration was a statistically significant variable (*p* = 0.004).

In model 9: The clinical and MRI variables explained almost 17% of total errors produced in WCST, and age (*p* = 0.012) and disease duration (*p* = 0.02) were statistically significant variables.

In model 10: The clinical and MRI variables explained over 16% of the WCST percentage of conceptual responses. The clinical variables, age (*p* = 0.014) and disease duration (*p* = 0.04), were statistically significant.

In model 11: The clinical and MRI variables explained about 10% of the total correct BRT score, and age was a statistically significant variable (*p* = 0.016).

In model 12: The clinical and MRI variables explained about 22% of the EDSS score, and education duration was a statistically significant variable *p* < 0.001.

In model 13: The clinical and MRI variables explained about 34% of the total correct 9HPT score, while education (*p* = 0.007), gender (0.011), and WM hypointensities volume (0.031) were statistically significant variables.

Additionally, we performed a univariate linear regression statistical analysis for groups CI and CN (Supplement 4) revealing that PSMD was a significant contributor (*p* = 0.036) to semantic fluency deficit in the CI subgroup. PSMD was also a statistically significant (*p* < 0.001) contributor to the 9HPT results in the CN subgroup. Other significant contributors in 9HPT in the CN subgroup were WM hypointensities (*p* < 0.001) and NBV (*p* = 0.002). In the CN subgroup, the regression analysis showed a significant relevance of NBV (*p* = 0.009) and WM hypointensties (*p* = 0.049) for the severity of disability (EDSS).

## 4 DISCUSSION

In this study, we have used a selection of neuropsychological tests to identify their associations with PSMD in RRMS patients. The results of our study show that PSMD and WM hypointensities were significant contributors to the CVLT score (word list A generation). This result is consistent with the findings of other authors (Vinciguerra et al., 2020) evaluating PSMD as a radiological marker of cognitive dysfunction in RRMS. CVLT assesses: Learning ability, information processing capacity, attention, and memory (Elwood, 1995), revealing abnormality in many functional networks including frontal‐limbic connections (Rao et al., 1984), and the thalamic‐hippocampal‐prefrontal circuit (Kern et al., 2015). Given that PSMD reveals the multisite disconnectivity of WM tracts, we could explain the association with CVLT processing failure. PSMD, as a parameter of the average magnitude of water molecular diffusion in the center of common WM tracts, provides information on individual variations in WM microstructure, and could better characterize the pathological process in RRMS rather than simple quantifying measures of WM LV and brain volume (Deary et al., 2019). We currently reveal PSMD is a significant contributor to semantic fluency (Supplement 4) in the CI subgroup. Semantic fluency is associated with semantic stock and the property of grouping named objects into categories (Barois et al., 2021). Structural and functional studies showed that semantic fluency is related to frontal and temporal structures, especially the cingulate cortex, medial supplementary motor area, left inferior gyrus, left posterior temporal lobe, and left inferior parietal lobe (Whiteside et al., 2016). VFT performance is associated with both executive function and language functioning (Whiteside et al., 2016). VFT is a reliable tool also in severe forms of MS and was established as an effective tool in assessing cognitive impairment in the MS population, with sensitivity and specificity, respectively, of 80.6% and 97.2% (Barois et al., 2021; Negreiros et al., 2008). We found currently no studies using VFT and diffusion MRI metrics in the MS population to compare results. This finding prompts us to further explore the association between verbal deficit in MS and widespread WM alterations.

Contrary to the aforementioned Vinciguerra et al.’s, 2020 study, in our cohort the main contributor of cognitive performance was education duration. This significant factor explained results achieved in such cognitive domains as: Processing speed (SDMT), attention (CCT, SDMT), semantic verbal fluency (VFT), and language learning processes and memory (CVLT‐list B). Previous studies on MS suggested that educational attainment (as assessed by years of education) reduces the negative effect of structural damage on cognition in MS (Pinter et al., 2014). The protective effect of education, as a way of developing a cognitive reserve in MS was also highlighted in recent studies (Sumowski et al., 2009). Our study extends this consideration to almost all cognitive domains. Prior studies presented a high relevance of PSMD for the SDMT score; however, we did not observe such a robust association, perhaps due to the heterogeneity of our RRMS population.

We also used WCST to evaluate executive function and in our cohort a significant contributor was age. With regard to our findings, a number of previous studies proved the association between WCST and age (Rhodes, 2004). Impaired performance on WCST (Kopp et al., 2021) has previously also been associated with focal lesions involving prefrontal lobe structures (Arnett et al., 1994). Therefore, the lack of a correlation between PSMD and WCST results could be explained by the fact that we used radiological markers of global WM deterioration (PSMD) and brain atrophy (normalized brain volume [NBV]). Another observation is that in the tested models, PASAT, contrary to SDMT, was not associated with any of the applied clinical and MRI variables, perhaps due to the lower sensitivity of PASAT, compared to SDMT in the assessment of cognitive efficiency (Sumowski et al., 2018).

In summary, we chose, for the regression models, MR variables such as PSMD, NBV, and WM hypointensities, which seem to reflect the pathological processes present in RRMS demyelination and brain atrophy (Wang et al., 2015). In our study, we observed that higher PSMD correlated with poorer performance. All mentioned markers were assumed to contribute to CI in the MS population. However, in our MS population, heterogenic in terms of disability and disease duration, educational attainment elucidated CI in the majority of evaluated cognitive domains. The results corroborate the observed lack of clear association between T1 lesion load, brain atrophy, and disability in RRMS in previous studies (Kolasa et al., 2019). In line with these results is the important role of regional cortical and subcortical GM atrophy underpinning cognitive deficit in MS (Petracca et al., 2021), which was omitted in the current study. Moreover, focal disruption of crucial functional brain networks due to axonal transection or demyelination could be critical for particular cognitive dysfunction (Arnett et al. 1994; da Silva et al., 2020; Tewarie et al., 2014). In our study, PSMD lost its significance as a disability contributor in the regression models of EDSS (Supplements 2,4). The regression analysis in subgroups revealed the major contributors for EDSS: NBV and WM hypointensities in the CN subgroup. Our finding is consistent with Vinciguerra et al.’s (2019) study , which also did not find the relevance of PSMD for global disability metrics (EDSS) and Kolasa et al.’s (2019) study , which did not reveal a significant association between baseline DTI parameters and EDSS at the baseline and after a year of follow‐up. It is noteworthy that PSMD, WM hypointensities, and NBV are significantly correlated with 9HPT in the CN subgroup. This association of PSMD and upper extremity function was not revealed in previous studies on the MS population (Vinciguerra et al., 2019). Further studies are needed to estimate PSMD relevance as a potential marker of disability in MS.

Our study corroborates and broadens previous findings of PSMD ability for evaluating microstructural WM alteration underlying cognitive and physical disability in MS. This study highlights the importance of cognitive reserve in reducing cognitive dysfunction caused by the structural damage of WM and GM in MS. In line with our findings, an essential effort should be made to develop cognitive reserve in the MS population. Similarly to other authors (Kolasa et al., 2019; Vinciguerra et al., 2020), our study is limited by a small RRMS cohort.

Other limitations of this study include its cross‐sectional character and lack of control group. Moreover, in our cohort, there were patients with quite a heterogeneity of disease duration, disability, and age. MRI limitations include the anistoropic voxel size and head movement, which can alter the DTI and volumetric measurements. Although the movement was taken into account in the analysis, it can still alter the metrics. Irrespective of the above‐mentioned limitations, our work sheds light on cognitive impairment contributors in the RRMS population.

There is still a demand for future research into novel MR markers identifying subjects at risk of cognitive deterioration.

## 5 CONCLUSIONS

We investigated PSMD as a new parameter of WM microstructure damage that contributes to complex cognitive tasks, CVLT, and semantic fluency. PSMD proved also to be a relevant marker of upper extremity dysfunction. In the studied population, in the majority of cognitive domains, educational attainment was a significant contributor of CI. Currently, the applicability of diffusion tensor MRI markers in the routine evaluation of cognitive dysfunction remains questionable due to still insufficient empirical data.

## CONFLICT OF INTEREST

The authors declare that there is no conflict of interest that could be perceived as prejudicing the impartiality of the research reported.

## FUNDING INFORMATION

This study was funded from the R&D ‐ dedicated internal resources of the Medical University of Gdansk.

## AUTHOR CONTRIBUTIONS

*The conception and design of the work, and the receipt of funding for the study*: Bartosz Karaszewski and Magdalena Chylińska; *Structure of team work*: Bartosz Karaszewski; *Data analysis and the interpretation, drafting and writing of the article*: Magdalena Chylińska; *Neurological assessment*: Magdalena Chylińska; *Neuropsychological assessment*: Jakub Komendziński.; *Statistical analysis*: Adam Wyszomirski.; *MR data acquisition and analysis*: Agnieszka Sabisz; *Critical revision of the interpretation and of the text*: Bartosz Karaszewski, Edyta Szurowska.

### PEER REVIEW

The peer review history for this article is available at https://publons.com/publon/10.1002/brb3.2591

## Supporting information

Supporting information1

Supporting information2

Supporting information3

Supporting information4

## Data Availability

The data that support the findings of this study are available from the corresponding author upon reasonable request.
