# Supplementary material for: Skeletonized mean diffusivity and neuropsychological performance in relapsing‐remitting multiple sclerosis
Source: Brain Behav. 2022 May 13;12(6):e2591. doi: 10.1002/brb3.2591 (PMC9226842; doi:10.1002/brb3.2591)
Supplement: Supplementary file 1 — Supporting information1 [file BRB3-12-e2591-s004.docx]

**2.3 Neuropsychological assessments**

Blinded to clinical and MRI data, experienced neuropsychologist (JK) performed neuropsychological assessment of all enrolled patients.
All patients performed the cognitive tests in the same order as listed below:
**Symbol Digit Modalities Test (SDMT**) in a written version was performed, each participant was given ninety seconds to pair specific numbers with given abstract symbols. The score was evaluated as a number of correct responses. Test assess attention, visual scanning, memory, mental flexibility and information processing speed [Smith A., 1982; Vogel A. et al., 2012].

**The California Verbal Learning Test (CVLT**) [Dean C. Delis et. al., 2000] in the polish adaptation [Łojek E. and Stańczak J., 2010] was performed. CVLT consists of three word lists: List A, List B, and the word list for recognition. Each Lists A and B contain 16 words that are categorically related to things that can be bought in the supermarket. The list of words to recognize includes 44 stimuli covering the entire List A, some words from List B, and other distractive words that meet certain conditions (e.g., phonetically close to words from List A). The respondent learns List A by repeating it five times immediately after the presentation, then recall it after a short delay (presentation of List B), after a long delay (20 minutes during which the respondent performs non-verbal tests) and recognizes it from 44 words on the recognition list. 21 CVLT performance indicators are quantified. This test allows the assessment of language learning processes, processes of extracting material from memory and recognition [Łojek J. and Stańczak E., 2010].

**Wisconsin Card Sorting Test (WCST)** [Heaton R.K et al., 1993] in polish adaptation [Jaworowska A., 2002].It consists of two decks of 64 cards each. The subject’s task is to match each card in the deck to one of the four reference cards. Test requires the participant to sort a set of cards according to implicit rules and based on the limited corrective feedback provided by the examiner, the participant’s responses can be analyzed to produce separate indices of sources of difficulty on the test [Jones C.R.G., 2013]. Test assess executive function in conceptual reasoning ability, controlling.

**Benton Visual Retention Test (BVRT)** in Polish adaptation of Jaworowska A. was performed. This is a tool designed by Arut L. Benton. The test consists of a notebook in which 10 patterns containing one or more geometric figures were printed on cardboard boxes. The patient's task is to redraw or draw from memory presented patterns. In order to enable multiple measurements with this tool, each test booklet contains three alternative test versions (C, D, E). The author of the Polish standardization studies verified the equivalence of test versions on the Polish population. The data presented in literature indicate that, depending on the age of the test group, these versions are characterized by different levels of difficulty. Each version of the test can be used with one of four methods of stimulus presentation. For the purpose of the study method A was used. This method consists of a 10-second presentation of the material. Then the patient after covering the viewed pattern has to immediately restore it from memory. The test is used to examine memory and visual perception [Jaworowska A, 2013].

**Color Trails Test (CTT)** is a new version of the Trail Making Test-TMT (Trail Making Test-TMT) [D’Elia et al.,1996]. Test is a new version of the Trail Making Test-TMT (Trail Making Test-TMT) which is widely used in diagnostics and research. This tool consists of two test sheets marked CTT1 and CTT2. An CTT- 1 test sheet has a sequence of numbers in the range from 1-25. All odd numbers are placed in pink circles, while all even numbers are printed in yellow circles. The patient's task is to connect as quickly as possible all numbers in ascending order. The researcher notes the time obtained by the patient as well as additional indicators such as the number of errors made in combining consecutive numbers, the number of so-called "almost errors" (incorrect reactions that were immediately corrected by the patient without the researcher's intervention) and the number of hints that were given to the patient. The tester is supposed to give a hint by indicating the next number if the patient does not make another call within 10 seconds. On the second sheet of this tool (CTT-2), each of the numbers from 1 to 25 is printed in both pink and yellow circles. This time the task of the patient is to connect the digits in ascending order, taking into account the color variations. The researcher writes down all the indicators that have already been mentioned during the first part of the test. An additional indicator included in this part of the test is the number of erroneous color combinations (it is a situation when a patient combines two circles of one color in sequence). Both parts of the test are designed to examine processes related to attention and executive functions (especially CTT-2 part) . The performance of this test requires the patient to engage such processes as: intentional material search, sequential information processing, attention maintenance, attention shiffting as well as monitoring his own behavior [Łojek E. and Stańczak J., 2012].

**Verbal Fluency Test (VFT)** [Piskunowicz M. et al., 2013], phonological fluency was tested giving patients 60 seconds to generate words starting with „k”. These words could not be the proper names. Semantic fluency assessment was performed asking patient to generate names of animals. Scoring is based on total number of words correctly generated. Both tests measure rapid retrival of verbal material [Vlaar AMM and Wade DT, 2003].

**Beck Depression Inventory-II (BDI-II)** in polish version of questionary was used. It is one of the most commonly used screening and self-descriptive tools to assess the severity of depressive disorders [Beck AT. Et al., 1996].
