# Supplementary material for: Skeletonized mean diffusivity and neuropsychological performance in relapsing‐remitting multiple sclerosis
Source: Brain Behav. 2022 May 13;12(6):e2591. doi: 10.1002/brb3.2591 (PMC9226842; doi:10.1002/brb3.2591)
Supplement: Supplementary file 2 — Supporting information2 [file BRB3-12-e2591-s002.docx]

Multivariate linear regression analysis.

**Model 1** Multivariate linear regression for Symbol Digit Modalities Test (SDMT).

| SDMT | Estimate  (Beta coefficient) | Lower 95%CI | Upper 95%CI | P-value | Adjusted R^2^ |
| --- | --- | --- | --- | --- | --- |
| Education (years) | 1.803 | 0.849 | 2.757 | **< 0.001** | **0.366** |
| Age (years) | 0.026 | -0.277 | 0.329 | 0.866 |  |
| Disease duration (years) | 0.151 | -0.343 | 0.645 | 0.543 |  |
| Gender, female | 4.527 | -0.398 | 9.453 | 0.071 |  |
| PSMD | -10175.409 | -76535.080 | 56184.262 | 0.760 |  |
| WM hypointensities | -1322.165 | -3130.465 | 486.135 | 0.149 |  |
| NBV | 55.992 | -23.594 | 135.579 | 0.165 |  |
| Abbreviations: SDMT, Symbol Digit Modalities Test, PSMD, peak width of skeletonized mean diffusivity, WM, white matter hypointensities normalized to estimated total intracranial volume, NBV, Normalized Brain Volume | | | | | |

**Model 2** Multivariate linear regression for Paced Auditory Serial Additive Test (PASAT)

| PASAT | Estimate  (Beta coefficient) | Lower 95%CI | Upper 95%CI | P-value | Adjusted  R^2^ |
| --- | --- | --- | --- | --- | --- |
| Education (years) | 0.599 | -0.439 | 1.637 | 0.253 | **0.022** |
| Age (years) | 0.061 | -0.269 | 0.391 | 0.713 |  |
| Disease duration (years) | -0.335 | -0.873 | 0.202 | 0.217 |  |
| Gender, female | 0.348 | -5.011 | 5.707 | 0.897 |  |
| PSMD | 26467.587 | -45739.664 | 98674.839 | 0.467 |  |
| WM hypointensities | -845.124 | -2812.770 | 1122.522 | 0.394 |  |
| NBV | 49.243 | -37.357 | 135.843 | 0.260 |  |
| Abbreviations: PASAT, Paced Auditory Serial Additive Test, PSMD, peak width of skeletonized mean diffusivity, WM, white matter hypointensities normalized to estimated total intracranial volume, NBV, Normalized Brain Volume | | | | | |

###

**Model 3** Multivariate linear regression for VFT -phonological fluency.

| VFT- phonological fluency | Estimate  (Beta coefficient) | Lower 95%CI | Upper 95%CI | P-value | Adjusted R^2^ |
| --- | --- | --- | --- | --- | --- |
| Education (years) | 0.494 | -0.050 | 1.039 | 0.075 | **0.023** |
| Age (years) | 0.158 | -0.015 | 0.331 | 0.072 |  |
| Disease duration (years) | -0.101 | -0.383 | 0.181 | 0.475 |  |
| Gender female | 1.202 | -1.610 | 4.014 | 0.396 |  |
| PSMD | -13740.962 | -51628.598 | 24146.675 | 0.471 |  |
| WM hypointensities | 398.429 | -634.008 | 1430.866 | 0.444 |  |
| NBV | 5.670 | -39.770 | 51.109 | 0.804 |  |
| Abbreviations: VFT, verbal fluency test, PSMD, peak width of skeletonized mean diffusivity, WM, white matter hypointensities normalized to estimated total intracranial volume, NBV, Normalized Brain Volume | | | | | |

###

**Model 4** Multivariate linear regression for VFT - semantic fluency.

| VFT - semantic fluency. | Estimate (Beta coefficient) | Lower 95%CI | Upper 95%CI | P-value | Adjusted R^2^ |
| --- | --- | --- | --- | --- | --- |
| Education (years) | 1.032 | 0.453 | 1.611 | **<0.001** | **0.134** |
| Age (years) | 0.151 | -0.033 | 0.335 | 0.106 |  |
| Disease duration (years) | -0.008 | -0.308 | 0.292 | 0.959 |  |
| Gender female | 0.104 | -2.887 | 3.095 | 0.945 |  |
| PSMD | -27118.925 | -67421.283 | 13183.434 | 0.184 |  |
| WM hypointensities | 183.490 | -914.749 | 1281.728 | 0.740 |  |
| NBV | -14.401 | -62.737 | 33.934 | 0.554 |  |
| Abbreviations: VFT, verbal fluency test, PSMD, peak width of skeletonized mean diffusivity, WM, white matter hypointensities normalized to estimated total intracranial volume, NBV, Normalized Brain Volume. | | | | | |

###

**Model 5** Multivariate linear regression for Color Trail Test 1 (CTT1).

| CTT 1 time | Estimate  (Beta coefficient) | Lower 95%CI | Upper 95%CI | P-value | Adjusted R^2^ |
| --- | --- | --- | --- | --- | --- |
| Education (years) | -1.708 | -3.230 | -0.187 | **0.028** | **0.236** |
| Age (years) | 0.184 | -0.300 | 0.667 | 0.451 |  |
| Disease duration (years) | 0.130 | -0.658 | 0.918 | 0.743 |  |
| Gender female | -6.530 | -14.386 | 1.327 | 0.102 |  |
| PSMD | 57685.448 | -48169.162 | 163540.058 | 0.280 |  |
| WM hypointensities | 1610.194 | -1274.343 | 4494.730 | 0.269 |  |
| NBV | 112.312 | -14.641 | 239.266 | 0.082 |  |
| Abbreviations: CCT1, Color Trail Test 1 , PSMD, peak width of skeletonized mean diffusivity, WM, white matter hypointensities normalized to estimated total intracranial volume, NBV, Normalized Brain Volume. | | | | | |

###

**Model 6** Multivariate linear regression for Color Trail Test 2 (CTT2).

| CCT2 | Estimate  (Beta coefficient) | Lower 95%CI | Upper 95%CI | P-value | Adjusted R^2^ |
| --- | --- | --- | --- | --- | --- |
| Education (years) | -3.090 | -5.473 | -0.707 | **0.012** | **0.200** |
| Age (years) | -0.108 | -0.865 | 0.649 | 0.778 |  |
| Disease duration (years) | 0.190 | -1.044 | 1.424 | 0.759 |  |
| Gender female | -2.745 | -15.050 | 9.559 | 0.657 |  |
| PSMD | 37637.279 | -128154.827 | 203429.384 | 0.652 |  |
| WM hypointensities | 2742.313 | -1775.519 | 7260.145 | 0.230 |  |
| NBV | -25.841 | -224.678 | 172.997 | 0.796 |  |
| Abbreviations: CCT2, Color Trail Test 2, PSMD, peak width of skeletonized mean diffusivity, WM, white matter hypointensities normalized to estimated total intracranial volume, NBV, Normalized Brain Volume. | | | | | |

**Model 7** Multivariate linear regression for CVLT – list A

| CVLT – list A | Estimate  (Beta coefficient) | Lower 95%CI | Upper 95%CI | P-value | Adjusted R^2^ |
| --- | --- | --- | --- | --- | --- |
| Education (years) | 1.356 | 0.574 | 2.137 | **< 0.001** | **0.320** |
| Age (years) | -0.057 | -0.305 | 0.191 | 0.649 |  |
| Disease duration (years) | -0.586 | -0.991 | -0.182 | **0.005** |  |
| Gender female | 2.704 | -1.329 | 6.738 | 0.185 |  |
| PSMD | -55190.239 | -109539.863 | -840.616 | **0.047** |  |
| WM hypointensities | 1757.768 | 276.741 | 3238.794 | **0.021** |  |
| NBV | 23.190 | -41.992 | 88.373 | 0.480 |  |
| Abbreviations: CVLT, The California Verbal Learning Test, PSMD, peak width of skeletonized mean diffusivity, WM, white matter hypointensities normalized to estimated total intracranial volume, NBV, Normalized Brain Volume | | | | | |

**Model 8**. Multivariate linear regression for CVLT list B.

| CVLT list B | Estimate  (Beta coefficient) | Lower 95%CI | Upper 95%CI | P-value | Adjusted R^2^ |
| --- | --- | --- | --- | --- | --- |
| Education (years) | 0.243 | 0.082 | 0.404 | **0.004** | **0.106** |
| Age (years) | -0.003 | -0.054 | 0.048 | 0.908 |  |
| Disease duration (years) | 0.021 | -0.063 | 0.104 | 0.623 |  |
| Gender female | -0.022 | -0.853 | 0.809 | 0.958 |  |
| PSMD | -6802.830 | -17999.692 | 4394.033 | 0.229 |  |
| WM hypointensities | 211.435 | -93.679 | 516.549 | 0.171 |  |
| NBV | 5.408 | -8.020 | 18.837 | 0.424 |  |
| Abbreviations: CVLT, The California Verbal Learning Test, PSMD, peak width of skeletonized mean diffusivity, WM, white matter hypointensities normalized to estimated total intracranial volume, NBV, Normalized Brain Volume | | | | | |

**Model 9** Multivariate linear regression of WCST, total errors.

| **WCST, total errors** | Estimate  (Beta coefficient) | Lower 95%CI | Upper 95%CI | P-value | Adjusted R^2^ |
| --- | --- | --- | --- | --- | --- |
| Education (years) | -1.284 | -3.285 | 0.717 | 0.205 | **0.168** |
| Age (years) | 0.823 | 0.187 | 1.459 | **0.012** |  |
| Disease duration (years) | -1.235 | -2.271 | -0.199 | **0.020** |  |
| Gender female | 4.596 | -5.736 | 14.929 | 0.378 |  |
| PSMD | -18956.122 | -158172.986 | 120260.742 | 0.787 |  |
| WM hypointensities | 2112.519 | -1681.138 | 5906.176 | 0.270 |  |
| NBV | 38.129 | -128.836 | 205.095 | 0.650 |  |
| Abbreviations: WCST, Wisconsin Card Sorting Test, PSMD, peak width of skeletonized mean diffusivity, WM, white matter hypointensities normalized to estimated total intracranial volume, NBV, Normalized Brain Volume. | | | | | |
|  | | | | | |

**Model 10** Multivariate linear regression of WCST, percentage of correct responses.

| **WCST, percentage of correct responses** | Estimate  (Beta coefficient) | Lower 95%CI | Upper 95%CI | P-value | Adjusted R^2^ |
| --- | --- | --- | --- | --- | --- |
| Education (years) | 1.083 | -0.716 | 2.883 | 0.234 | **0.161** |
| Age (years) | -0.725 | -1.296 | -0.153 | **0.014** |  |
| Disease duration (years) | 0.979 | 0.048 | 1.911 | **0.040** |  |
| Gender female | -5.675 | -14.964 | 3.615 | 0.227 |  |
| PSMD | 11682.069 | -113482.299 | 136846.437 | 0.853 |  |
| WM hypointensities | -1665.311 | -5076.038 | 1745.416 | 0.333 |  |
| NBV | -33.390 | -183.502 | 116.722 | 0.658 |  |
| Abbreviations: WCST, Wisconsin Card Sorting Test, PSMD, peak width of skeletonized mean diffusivity, WM, white matter hypointensities normalized to estimated total intracranial volume, NBV, Normalized Brain Volume | | | | | |
|  | | | | | |

**Model 11** Multivariate linear regression of BRT, total correct endpoint.

| BRT, total correct | Estimate  (Beta coefficient) | Lower 95%CI | Upper 95%CI | P-value | Adjusted R^2^ |
| --- | --- | --- | --- | --- | --- |
| Education (years) | 0.097 | -0.046 | 0.241 | 0.180 | **0.106** |
| Age (years) | -0.057 | -0.102 | -0.011 | **0.016** |  |
| Disease duration (years) | 0.010 | -0.064 | 0.084 | 0.786 |  |
| Gender female | -0.062 | -0.803 | 0.678 | 0.867 |  |
| PSMD | -3264.724 | -13244.151 | 6714.703 | 0.516 |  |
| WM hypointensities | 24.249 | -247.690 | 296.188 | 0.859 |  |
| NBV | -4.089 | -16.058 | 7.879 | 0.497 |  |
| Abbreviations: BVRT, Benton Visual Retention Test, PSMD, peak width of skeletonized mean diffusivity, WM, white matter hypointensities normalized to estimated total intracranial volume, NBV, Normalized Brain Volume | | | | | |
|  | | | | | |

**Model 12** Multivariate linear regression of EDSS.

| **EDSS** | Estimate  (Beta coefficient) | Lower 95%CI | Upper 95%CI | P-value | Adjusted R^2^ |
| --- | --- | --- | --- | --- | --- |
| Education (years) | -0.158 | -0.248 | -0.067 | **< 0.001** | 0.216 |
| Age (years) | -0.001 | -0.029 | 0.028 | 0.967 |  |
| Disease duration (years) | 0.037 | -0.010 | 0.083 | 0.123 |  |
| Gender female | 0.118 | -0.349 | 0.584 | 0.616 |  |
| PSMD | -2361.304 | -8645.908 | 3923.301 | 0.456 |  |
| WM hypointensities | 69.019 | -102.236 | 240.275 | 0.424 |  |
| Brain vol. [normalized to eTIV] | -4.686 | -12.224 | 2.851 | 0.219 |  |
| Abbreviations: EDSS, Expanded Disability Status Scale, PSMD, peak width of skeletonized mean diffusivity, WM , white matter, vol., volume, eTIV, estimated total intracranial volume. | | | | | |

**Model 13** Multivariate linear regression of 9-HPT

| **9-HPT** | Estimate  (Beta coefficient) | Lower 95%CI | Upper 95%CI | P-value | Adjusted R^2^ |
| --- | --- | --- | --- | --- | --- |
| Education (years) | -0.652 | -1.116 | -0.189 | 0.007 | 0.344 |
| Age (years) | -0.046 | -0.193 | 0.101 | 0.536 |  |
| Disease duration (years) | 0.097 | -0.143 | 0.337 | 0.421 |  |
| Gender female | -3.145 | -5.538 | -0.753 | 0.011 |  |
| PSMD | -4230.089 | -36469.806 | 28009.628 | 0.794 |  |
| WM hypointensities | 970.859 | 92.328 | 1849.391 | 0.031 |  |
| Brain vol. [normalized to eTIV] | -12.137 | -50.803 | 26.529 | 0.533 |  |
| Abbreviations: EDSS, Expanded Disability Status Scale, PSMD, peak width of skeletonized mean diffusivity, WM , white matter, vol., volume, eTIV, estimated total intracranial volume. | | | | | |
