# Supplementary material for: Skeletonized mean diffusivity and neuropsychological performance in relapsing‐remitting multiple sclerosis
Source: Brain Behav. 2022 May 13;12(6):e2591. doi: 10.1002/brb3.2591 (PMC9226842; doi:10.1002/brb3.2591)
Supplement: Supplementary file 3 — Supporting information3 [file BRB3-12-e2591-s003.docx]

**Tabel 1.** Comparison of radiological and clinical parameters in MS subgroups

|  | CI (N=37) | CN (N=36) | p value |
| --- | --- | --- | --- |
| **PSMD x 10^-4^** |  |  | 0.262 |
| Mean (SD) | 2.728 (0.666) | 2.915 (0.734) |  |
| Median (Q1,Q3) | 2.612 (2.229, 3.034) | 2.708 (2.431, 3.261) |  |
| **WM hypointensities** |  |  | 0.158 |
| Mean (SD) | 0.003 (0.002) | 0.004 (0.003) |  |
| Median (Q1,Q3) | 0.002 (0.001, 0.004) | 0.003 (0.002, 0.004) |  |
| **NBV** |  |  | 0.354 |
| Mean (SD) | 0.754 (0.030) | 0.749 (0.040) |  |
| Median (Q1,Q3) | 0.758 (0.739, 0.770) | 0.753 (0.726, 0.768) |  |
| **Education, y** |  |  | 0.006 |
| Mean (SD) | 14.162 (2.641) | 15.861 (2.031) |  |
| Median (Q1,Q3) | 13.000 (12.000, 17.000) | 17.000 (15.000, 17.000) |  |
| **Age, y** |  |  | 0.345 |
| Mean (SD) | 38.784 (9.080) | 37.389 (9.259) |  |
| Median (Q1,Q3) | 43.000 (30.000, 45.000) | 39.000 (29.000, 42.250) |  |
| Abbreviations: MS, multiple sclerosis, CI, cognitive impaired, CN, cognitive normal, PSMD, peak width of skeletonized mean diffusivity, SD - standard deviation, Q1 - the first quartile, Q3 - the third quartile, WM, white matter hypointensities normalized to estimated total intracranial volume, NBV, Normalized Brain Volume to eTIV - estimated total intracranial volume, y, years. | | | |

###

**Tabel 2.** Comparison of clinical parameters in MS subgroups

|  | CI (N=37) | CN (N=36) | p-value |
| --- | --- | --- | --- |
| **SDMT** |  |  | < 0.001 |
| Mean (SD) | 39.000 (11.218) | 54.167 (7.962) |  |
| Median (Q1,Q3) | 40.000 (32.000, 48.000) | 53.500 (48.750, 58.000) |  |
| **PASAT** |  |  | 0.008 |
| Mean (SD) | 41.027 (11.959) | 48.500 (6.955) |  |
| Median (Q1,Q3) | 42.000 (36.000, 51.000) | 48.000 (45.000, 55.000) |  |
| **Verbal Fluency** |  |  | < 0.001 |
| Mean (SD) | 13.486 (4.647) | 18.694 (4.714) |  |
| Median (Q1,Q3) | 13.000 (10.000, 16.000) | 18.000 (15.750, 22.000) |  |
| **Semantic Fluency** |  |  | < 0.001 |
| Mean (SD) | 18.108 (4.115) | 24.333 (6.127) |  |
| Median (Q1,Q3) | 17.000 (15.000, 20.000) | 25.000 (21.500, 28.250) |  |
| **CTT1 time** |  |  | < 0.001 |
| Mean (SD) | 47.811 (17.790) | 32.250 (11.175) |  |
| Median (Q1,Q3) | 42.000 (35.000, 56.000) | 28.500 (24.000, 38.500) |  |
| **CTT2 time** |  |  | < 0.001 |
| Mean (SD) | 96.973 (26.088) | 68.861 (16.204) |  |
| Median (Q1,Q3) | 93.000 (80.000, 108.000) | 65.500 (57.750, 77.750) |  |
| **CVLT list-A** |  |  | < 0.001 |
| Mean (SD) | 48.757 (8.764) | 58.000 (6.920) |  |
| Median (Q1,Q3) | 49.000 (44.000, 56.000) | 58.000 (53.000, 62.500) |  |
| **WCST percentage of conceptual responses** |  |  | 0.005 |
| Mean (SD) | 60.930 (21.996) | 75.570 (11.870) |  |
| Median (Q1,Q3) | 69.400 (42.200, 81.100) | 77.900 (71.273, 85.700) |  |
| **WCST Number of achieved categories** |  |  | < 0.001 |
| Mean (SD) | 4.189 (1.970) | 5.806 (0.577) |  |
| Median (Q1,Q3) | 6.000 (2.000, 6.000) | 6.000 (6.000, 6.000) |  |
| **BVRT, correct total** |  |  | 0.002 |
| Mean (SD) | 7.135 (1.494) | 8.167 (1.183) |  |
| Median (Q1,Q3) | 7.000 (6.000, 8.000) | 8.000 (7.750, 9.000) |  |
| **BVRT, errors** |  |  | < 0.001 |
| Mean (SD) | 4.541 (2.652) | 2.500 (1.964) |  |
| Median (Q1,Q3) | 4.000 (3.000, 6.000) | 2.000 (1.000, 3.250) |  |
| **EDSS** |  |  | 0.366 |
| Mean (SD) | 2.284 (0.838) | 2.569 (1.122) |  |
| Median (Q1,Q3) | 2.000 (1.500, 3.000) | 2.500 (1.500, 3.000) |  |
| **9HPT** |  |  | 0.075 |
| Mean (SD) | 22.038 (7.604) | 23.692 (6.185) |  |
| Median (Q1,Q3) | 20.200 (17.900, 22.100) | 21.800 (19.300, 26.425) |  |
| Abbreviations: CI, cognitive impaired, CN, cognitive normal, SD - standard deviation, Q1 - the first quartile, Q3 - the third quartile SDMT - Symbol Digit Modalities Test, PASAT - Paced Auditory Serial Additive Test, VFT - Verbal Fluency Test, CTT - Color Trails Test, WCST - Wisconsin Card Sorting Test, BVRT - Benton Visual Retention Test, CVLT TOT - California Verbal Learning Test Total Recall, EDSS, Expanded Disability Status Scale , 9HPT - 9 Hole Peg Test | | | |
