# Supplementary material for: Skeletonized mean diffusivity and neuropsychological performance in relapsing‐remitting multiple sclerosis
Source: Brain Behav. 2022 May 13;12(6):e2591. doi: 10.1002/brb3.2591 (PMC9226842; doi:10.1002/brb3.2591)
Supplement: Supplementary file 4 — Supporting information4 [file BRB3-12-e2591-s001.docx]

**Tabel 1.** Univariate linear regression analysis for cognitive and clinical data in CN and CI subgroup of MS population

| Subgroups of MS population | **SDMT** | Estimate  (Beta coefficient) | Lower 95%CI | Upper 95%CI | p-value | R^2^ |
| --- | --- | --- | --- | --- | --- | --- |
| **CN** | PSMDx10^-4^ | 2.668 | -0.999 | 6.334 | 0.148 | 0.060 |
|  | WM hypointensities | 471.908 | -401.218 | 1345.035 | 0.280 | 0.034 |
|  | NBV | -80.245 | -143.779 | -16.712 | **0.015** | **0.162** |
|  | Education duration | 0.546 | -0.807 | 1.900 | 0.418 | 0.019 |
|  | Age | -0.177 | -0.470 | 0.116 | 0.228 | 0.042 |
| **CI** | PSMDx10^-4^ | 1.968 | -3.774 | 7.711 | 0.491 | 0.014 |
|  | WM hypointensities | 869.160 | -1123.038 | 2861.357 | 0.382 | 0.022 |
|  | NBV | -21.992 | -148.257 | 104.273 | 0.726 | 0.004 |
|  | Education duration | 1.996 | 0.709 | 3.283 | **0.003** | **0.221** |
|  | Age | -0.202 | -0.620 | 0.216 | 0.334 | 0.027 |

| Subgroups of MS population | **PASAT** | Estimate  (Beta coefficient) | Lower 95%CI | Upper 95%CI | p-value | R^2^ |
| --- | --- | --- | --- | --- | --- | --- |
| **CN** | PSMDx10^-4^ | -0.537 | -3.836 | 2.761 | 0.743 | 0.003 |
|  | WM hypointensities | -280.369 | -1050.259 | 489.520 | 0.464 | 0.016 |
|  | NBV | -35.210 | -94.589 | 24.169 | 0.237 | 0.041 |
|  | Education duration | 0.232 | -0.959 | 1.423 | 0.695 | 0.005 |
|  | Age | -0.088 | -0.348 | 0.172 | 0.496 | 0.014 |
| **CI** | PSMDx10^-4^ | 2.436 | -3.672 | 8.543 | 0.424 | 0.018 |
|  | WM hypointensities | 870.523 | -1256.218 | 2997.263 | 0.412 | 0.019 |
|  | NBV | -36.209 | -170.491 | 98.073 | 0.588 | 0.008 |
|  | Education duration | 0.605 | -0.935 | 2.145 | 0.431 | 0.018 |
|  | Age | -0.106 | -0.556 | 0.345 | 0.637 | 0.006 |

| Subgroups of MS population | **Phonological fluency** | Estimate  (Beta coefficient) | Lower 95%CI | Upper 95%CI | p-value | R^2^ |
| --- | --- | --- | --- | --- | --- | --- |
| CN | PSMDx10^-4^ | 0.770 | -1.452 | 2.993 | 0.486 | 0.014 |
|  | WM hypointensities | 233.828 | -285.788 | 753.444 | 0.367 | 0.024 |
|  | NBV | -3.704 | -44.777 | 37.370 | 0.856 | 0.001 |
|  | Education duration | 0.204 | -0.602 | 1.010 | 0.610 | 0.008 |
|  | Age | 0.029 | -0.148 | 0.207 | 0.738 | 0.003 |
| CI | PSMDx10^-4^ | -0.569 | -2.956 | 1.818 | 0.632 | 0.007 |
|  | WM hypointensities | -27.189 | -861.538 | 807.160 | 0.948 | 0.000 |
|  | NBV | -0.282 | -52.676 | 52.113 | 0.991 | 0.000 |
|  | Education duration | 0.371 | -0.219 | 0.961 | 0.211 | 0.044 |
|  | Age | 0.068 | -0.106 | 0.242 | 0.433 | 0.018 |

| Subgroups of MS  population | **Semantic fluency** | Estimate  (Beta coefficient) | Lower 95%CI | Upper 95%CI | p-value | R^2^ |
| --- | --- | --- | --- | --- | --- | --- |
| CN | PSMDx10^-4^ | -0.978 | -3.869 | 1.912 | 0.496 | 0.014 |
|  | WM hypointensities | -173.374 | -854.402 | 507.654 | 0.608 | 0.008 |
|  | NBV | 17.835 | -35.219 | 70.890 | 0.499 | 0.014 |
|  | Education duration | 0.808 | -0.205 | 1.822 | 0.114 | 0.072 |
|  | Age | -0.028 | -0.258 | 0.203 | 0.807 | 0.002 |
| CI | PSMDx10^-4^ | -2.137 | -4.127 | -0.147 | **0.036** | 0.120 |
|  | WM hypointensities | -718.711 | -1415.276 | -22.145 | **0.044** | 0.111 |
|  | NBV | 13.431 | -32.740 | 59.601 | 0.559 | 0.010 |
|  | Education duration | 0.404 | -0.113 | 0.920 | 0.122 | 0.067 |
|  | Age | -0.008 | -0.164 | 0.147 | 0.916 | 0.000 |
| Subgroups of MS population | **CTT 1 time** | Estimate  (Beta coefficient) | Lower 95%CI | Upper 95%CI | p-value | R^2^ |
| CN | PSMDx10^-4^ | -4.726 | -9.772 | 0.320 | 0.065 | 0.096 |
|  | WM hypointensities | -907.094 | -2113.298 | 299.111 | 0.136 | 0.064 |
|  | NBV | 108.582 | 18.809 | 198.355 | **0.019** | 0.151 |
|  | Education duration | 0.508 | -1.402 | 2.418 | 0.593 | 0.009 |
|  | Age | 0.167 | -0.249 | 0.584 | 0.421 | 0.019 |
| CI | PSMDx10^-4^ | 0.439 | -8.729 | 9.607 | 0.923 | 0.000 |
|  | WM hypointensities | -693.408 | -3879.223 | 2492.407 | 0.661 | 0.006 |
|  | NBV | 81.826 | -116.804 | 280.455 | 0.409 | 0.020 |
|  | Education duration | -2.171 | -4.359 | 0.018 | 0.052 | 0.104 |
|  | Age | 0.785 | 0.170 | 1.401 | **0.014** | 0.161 |

| Subgroups of MS population | **CTT2 time** | Estimate  (Beta coefficient) | Lower 95%CI | Upper 95%CI | p-value | R^2^ |
| --- | --- | --- | --- | --- | --- | --- |
| CN | PSMDx10^-4^ | -6.366 | -13.737 | 1.004 | 0.088 | 0.083 |
|  | WM hypointensities | -1184.629 | -2945.013 | 575.755 | 0.180 | 0.052 |
|  | NBV | 170.892 | 42.791 | 298.993 | **0.010** | 0.178 |
|  | Education duration | -0.566 | -3.340 | 2.208 | 0.681 | 0.005 |
|  | Age | 0.696 | 0.136 | 1.255 | **0.016** | 0.158 |
| CI | PSMDx10^-4^ | -2.751 | -16.164 | 10.662 | 0.680 | 0.005 |
|  | WM hypointensities | -1326.171 | -5988.785 | 3336.443 | 0.567 | 0.009 |
|  | NBV | 123.311 | -167.801 | 414.423 | 0.396 | 0.021 |
|  | Education duration | -2.872 | -6.115 | 0.372 | 0.081 | 0.084 |
|  | Age | 1.079 | 0.165 | 1.992 | **0.022** | **0.141** |

| Subgroups of MS population | **CVLT List A** | Estimate  (Beta coefficient) | Lower 95%CI | Upper 95%CI | p-value | R^2^ |
| --- | --- | --- | --- | --- | --- | --- |
| CN | PSMDx10^-4^ | 1.285 | -1.971 | 4.541 | 0.428 | 0.019 |
|  | WM hypointensities | 314.902 | -449.418 | 1079.222 | 0.408 | 0.020 |
|  | NBV | -3.220 | -63.538 | 57.098 | 0.914 | 0.000 |
|  | Education duration | 0.741 | -0.418 | 1.901 | 0.202 | 0.047 |
|  | Age | -0.087 | -0.346 | 0.171 | 0.497 | 0.014 |
| CI | PSMDx10^-4^ | -2.748 | -7.165 | 1.670 | 0.215 | 0.044 |
|  | WM hypointensities | -861.634 | -2407.336 | 684.068 | 0.265 | 0.035 |
|  | NBV | -3.734 | -102.545 | 95.077 | 0.939 | 0.000 |
|  | Education duration | 1.380 | 0.344 | 2.416 | **0.010** | **0.173** |
|  | Age | -0.294 | -0.609 | 0.022 | 0.067 | 0.093 |

| Subgroups of MS population | **CVLT List B** | Estimate  (Beta coefficient) | Lower 95%CI | Upper 95%CI | p-value | R^2^ |
| --- | --- | --- | --- | --- | --- | --- |
| CN | PSMDx10^4^ | 0.100 | -0.820 | 1.021 | 0.826 | 0.001 |
|  | WM hypointensities | 29.182 | -186.981 | 245.346 | 0.785 | 0.002 |
|  | NBV | -3.205 | -20.076 | 13.665 | 0.702 | 0.004 |
|  | Education duration | 0.170 | -0.158 | 0.497 | 0.300 | 0.032 |
|  | Age | -0.011 | -0.084 | 0.062 | 0.756 | 0.003 |
| CI | PSMDx10^4^ | -0.225 | -1.575 | 1.125 | 0.737 | 0.003 |
|  | WM hypointensities | -86.458 | -556.542 | 383.625 | 0.711 | 0.004 |
|  | NBV | -15.594 | -44.683 | 13.495 | 0.284 | 0.033 |
|  | Education duration | 0.347 | 0.028 | 0.666 | **0.034** | 0.122 |
|  | Age | -0.096 | -0.189 | -0.002 | 0.046 | 0.109 |

| Subgroups of MS population | **WCST percentage of conceptual responses** | Estimate  (Beta coefficient) | Lower 95%CI | Upper 95%CI | p-value | R^2^ |
| --- | --- | --- | --- | --- | --- | --- |
| CN | PSMDx10^-4^ | 1.385 | -4.232 | 7.003 | 0.619 | 0.007 |
|  | WM hypointensities | 150.025 | -1173.440 | 1473.491 | 0.819 | 0.002 |
|  | NBV | -36.354 | -139.059 | 66.350 | 0.477 | 0.015 |
|  | Education duration | -0.184 | -2.221 | 1.852 | 0.855 | 0.001 |
|  | Age | -0.082 | -0.528 | 0.363 | 0.709 | 0.004 |
| CI | PSMDx10^-4^ | -6.088 | -17.231 | 5.055 | 0.275 | 0.034 |
|  | WM hypointensities | -1172.01 | -5101.359 | 2757.340 | 0.549 | 0.010 |
|  | NBV | 30.892 | -216.907 | 278.690 | 0.802 | 0.002 |
|  | Education duration | 1.904 | -0.878 | 4.687 | 0.173 | 0.052 |
|  | Age | -1.188 | -1.912 | -0.463 | **0.002** | 0.240 |

|  |  |  |  |  |  |  |
| --- | --- | --- | --- | --- | --- | --- |
|  |  |  |  |  |  |  |
|  |  |  |  |  |  |  |

| Subgroups of MS population | **BENTON total corrects** | Estimate  (Beta coefficient) | | Lower 95%CI | Upper 95%CI | p-value | R^2^ |
| --- | --- | --- | --- | --- | --- | --- | --- |
| CN | PSMDx10^-4^ | | -0.386 | -0.932 | 0.160 | 0.160 | 0.057 |
|  | WM hypointensities | | -73.366 | -202.895 | 56.163 | 0.258 | 0.038 |
|  | NBV | | 7.383 | -2.606 | 17.372 | 0.142 | 0.062 |
|  | Education duration | | 0.172 | -0.022 | 0.366 | 0.080 | 0.087 |
|  | Age | | -0.041 | -0.084 | 0.001 | 0.054 | 0.105 |
| CI | PSMDx10^-4^ | | -0.591 | -1.334 | 0.151 | 0.115 | 0.070 |
|  | WM hypointensities | | -177.440 | -438.675 | 83.796 | 0.177 | 0.052 |
|  | NBV | | 2.643 | -14.176 | 19.462 | 0.752 | 0.003 |
|  | Education duration | | 0.029 | -0.165 | 0.223 | 0.766 | 0.003 |
|  | Age | | -0.075 | -0.125 | -0.024 | **0.005** | 0.207 |

| Subgroups of MS population | **BENTON total errors** | Estimate  (Beta coefficient) | | Lower 95%CI | Upper 95%CI | p-value | R^2^ |
| --- | --- | --- | --- | --- | --- | --- | --- |
| CN | PSMDx10^-4^ | | 0.563 | -0.349 | 1.475 | 0.218 | 0.044 |
|  | WM hypointensities | | 60.327 | -157.810 | 278.464 | 0.578 | 0.009 |
|  | NBV | | -7.011 | -23.958 | 9.935 | 0.406 | 0.020 |
|  | Education duration | | -0.260 | -0.585 | 0.065 | 0.113 | 0.072 |
|  | Age | | 0.077 | 0.008 | 0.146 | **0.030** | **0.132** |
| CI | PSMDx10^-4^ | | 0.941 | -0.387 | 2.270 | 0.159 | 0.056 |
|  | WM hypointensities | | 300.837 | -164.072 | 765.746 | 0.198 | 0.047 |
|  | NBV | | -4.592 | -34.454 | 25.271 | 0.757 | 0.003 |
|  | Education duration | | -0.057 | -0.401 | 0.287 | 0.740 | 0.003 |
|  | Age | | 0.152 | 0.067 | 0.238 | **< 0.001** | **0.272** |

| Subgroups of MS population | **EDSS** | estimate | CI.lower.estimate | CI.upper.estimate | p.value | R^2^ |
| --- | --- | --- | --- | --- | --- | --- |
| CN | PSMDx10^-4^ | 0.398 | -0.117 | 0.912 | 0.126 | 0.068 |
|  | WM hypointensities | 118.654 | 0.460 | 236.848 | **0.049** | **0.109** |
|  | NBV | -12.023 | -20.863 | -3.182 | **0.009** | **0.183** |
|  | Education duration | 0.086 | -0.105 | 0.276 | 0.367 | 0.024 |
|  | Age | 0.026 | -0.016 | 0.067 | 0.218 | 0.044 |
| CI | PSMDx10^-4^ | 0.255 | -0.168 | 0.678 | 0.229 | 0.041 |
|  | WM hypointensities | 49.069 | -100.439 | 198.577 | 0.510 | 0.013 |
|  | NBV | -1.947 | -11.370 | 7.477 | 0.678 | 0.005 |
|  | Education duration | -0.019 | -0.127 | 0.090 | 0.728 | 0.003 |
|  | Age | 0.002 | -0.030 | 0.034 | 0.893 | 0.001 |

| Subgroups of MS population | **9HPT** | Estimate  (Beta coefficient) | Lower 95%CI | Upper 95%CI | p.value | R^2^ |
| --- | --- | --- | --- | --- | --- | --- |
| CN | PSMDx10^-4^ | 4.988 | 2.620 | 7.357 | **< 0.001** | **0.350** |
|  | WM hypointensities | 1254.907 | 721.054 | 1788.759 | **< 0.001** | **0.402** |
|  | NBV | -78.181 | -124.710 | -31.652 | **0.002** | **0.255** |
|  | Education duration | 0.203 | -0.856 | 1.262 | 0.700 | 0.004 |
|  | Age | 0.075 | -0.156 | 0.306 | 0.514 | 0.013 |
| CI | PSMDx10^-4^ | 3.005 | -0.776 | 6.786 | 0.116 | 0.069 |
|  | WM hypointensities | 525.910 | -827.573 | 1879.392 | 0.436 | 0.017 |
|  | NBV | -51.827 | -135.704 | 32.050 | 0.218 | 0.043 |
|  | Education duration | -0.130 | -1.117 | 0.858 | 0.791 | 0.002 |
| \| Abbreviations: CI, cognitive impaired, CN, cognitive normal, SD - standard deviation, Q1, the first quartile, Q3, the third quartile, CI, confidence interval, R2, coefficient of determination, PSMD, peak width of skeletonized mean diffusivity, WM, white matter hypointensities normalized to estimated total intracranial volume, NBV, Normalized Brain Volume to eTIV - estimated total intracranial volume, SDMT, Symbol Digit Modalities Test, PASAT, Paced Auditory Serial Additive Test, VFT, Verbal Fluency Test, CTT, Color Trails Test, WCST, Wisconsin Card Sorting Test, BVRT, Benton Visual Retention Test, CVLT, California Verbal Learning Test, EDSS, Expanded Disability Status Scale , EDSS, Expanded Disability Status Scale, 9HPT, 9 Hole Peg Test, \| \| --- \| | | | | | | |

### 
